# Prognostic and Clinical Value of Interleukin 6 and CD45^+^CD14^+^ Inflammatory Cells with PD-L1^+^/PD-L2^+^ Expression in Patients with Different Manifestation of Ovarian Cancer

**DOI:** 10.1155/2020/1715064

**Published:** 2020-09-30

**Authors:** Iwona Wertel, Dorota Suszczyk, Anna Pawłowska, Monika Bilska, Agata Chudzik, Wiktoria Skiba, Roman Paduch, Jan Kotarski

**Affiliations:** ^1^Independent Laboratory of Cancer Diagnostics and Immunology, I Chair and Department of Oncological Gynaecology and Gynaecology, Medical University of Lublin, Staszica 16, Lublin 20-081, Poland; ^2^Independent Public Clinical Hospital No. 1, Medical University of Lublin, Staszica 16, Lublin 20-081, Poland; ^3^Students' Scientific Association, Independent Laboratory of Cancer Diagnostics and Immunology, I Chair and Department of Gynecologic Oncology and Gynecology, Medical University of Lublin, Staszica 16, Lublin 20-081, Poland; ^4^Department of Virology and Immunology, Maria Curie-Skłodowska University, Akademicka 19, 20-033 Lublin, Poland; ^5^I Chair and Department of Oncological Gynaecology and Gynaecology, Medical University of Lublin, Staszica 16, Lublin 20-081, Poland

## Abstract

Ovarian cancer (OC) is one of the deadliest gynecological cancers. Recent studies suggest a crucial role of inflammatory immune system cells in the progression and metastasis of OC. The understanding of inflammatory mechanisms is pivotal for the selection of a biomarker that allows the differentiation between malignant and benign tumors, monitoring the progression of the disease, and identification of patients that will respond to implemented treatment. Our study is aimed at evaluating the profile of IL-6 in the plasma and peritoneal fluid (PF) of patients with various clinical manifestations of OC (*n* = 78). We also examined the relationship between IL-6 and PD-L1/PD-L2 positive CD45^+^CD14^+^ inflammatory cell (MO/MA) levels in three OC environments (TME): peripheral blood (PB), PF, and tumor (TT) and their clinical and prognostic relevance in OC patients. The expression of PD-L1/PD-L2 molecules was analyzed by flow cytometry. The IL-6 levels were determined by ELISA. We found an elevated level of PD-L1/PD-L2 positive MO/MA in TT compared to PB (*p* < 0.0001). Significantly higher (*p* < 0.0001) levels of IL-6 were observed in PF of the OC patients than in the benign ovarian tumor group (*n* = 31). Additionally, we found higher IL-6 levels in PF than in the plasma of the OC patients. Interestingly, accumulation of IL-6 was observed in PF of patients with low-differentiated OC and correlated with worse prognosis. Moreover, we observed correlations between the level of IL-6 and CD45^+^CD14^+^ cells and between CD45^+^CD14^+^PD-L1^+^ cells and the IL-6 level in PF. For the first time, we discovered that the higher percentage of CD45^+^CD14^+^PD-L2^+^ cells in PF predicts better survival of OC patients. Our study suggests that CD45^+^CD14^+^PD-L2^+^ cells and IL-6 may be predictive biomarkers for OC patients. Understanding how the composition of TME changes during OC development and progression is a prerequisite for projecting new therapeutic strategies. Overall, further validation research is warranted.

## 1. Introduction

The prognosis for ovarian cancer (OC) patients is extremely poor. Its high mortality rate is a result of the lack of screening methods and the specific symptoms of the disease, which is related to the extreme heterogeneity of OC, including genomic, transcriptomic, proteomic, and, the very least known, immunologic aspects [1–3]. The clinical outcomes of OC patients depend on many factors, e.g., the International Federation of Gynecology and Obstetrics (FIGO) stage, differentiation grade (G), and histological type of the tumor [4–6]. The mechanisms of OC escape from immune surveillance, tissue invasion, and metastasis remain unexplained. It has been proved that OC is an immunogenic tumor which induces antitumor immune response found in different ovarian cancer microenvironments (TMEs), e.g., peripheral blood (PB), peritoneal fluid (PF), and tumor tissue (TT). However, the response is reinforced by locally occurring immune cells that induce strong inflammation and immunosuppression in TMEs. The tumor microenvironment in which OC develops has been described to be enriched with a broad spectrum of proinflammatory chemokines (CCL2, CCL4, and CXCL10), cytokines (e.g., IL-6, IL-8, IL-10, and IL-12), and factors (TNF-*α* and TGF-*β*), which have been shown to influence clinical disease status and prognosis [7–9]. Among these, IL-6 is of particular importance, because it exerts a wide variety of biological functions, particularly involved in the induction of inflammation and resultant carcinogenesis as well as immune suppression in patients with cancer [10, 11].

Interleukin 6 (IL-6) is a 26 kDa glycoprotein consisting of 184 amino acids [12]. The family of cytokines, that includes IL-6, utilizes receptors composed of two subunits: glycoprotein 130 (gp130) and a cytokine-specific *α* chain. IL-6 is produced by monocytes/macrophages (MO/MA) and many other cells, e.g., lymphocytes, fibroblasts, adipocytes, endothelial cells, and mesothelial cells [13]. An increase in its concentration is observed in autoimmune diseases, such as rheumatoid arthritis, Crohn's disease, Castleman disease, acute pancreatitis, bacterial infections, or sepsis [14, 15]. Increased serum levels of IL-6 have also been observed in patients with cancer, including breast, colorectal, stomach, and pancreatic cancers; malignant mesothelioma; and ovarian cancer [16–18].

It has been shown that IL-6 is responsible for acute phase responses, T lymphocyte stimulation, and B lymphocyte proliferation and differentiation in neoplastic diseases. It is involved in mechanisms responsible for neoplastic cachexia and development of osteoporosis [19]. IL-6 seems to be one of the determinants of chemotherapy resistance in OC patients with ascites. Furthermore, IL-6 inhibits the apoptosis of ovarian cancer cells contributing to tumor growth and induces vascular endothelial growth factor- (VEGF-) mediated angiogenesis. According to recent study findings, IL-6 and other proinflammatory cytokines of this family, including oncostatin M, directly stimulate the invasion of cancer cells through basement membrane degradation due to metalloproteinase (MMP) overexpression, induce epithelial-mesenchymal transition of ovarian epithelial cells, and increase resistance to chemotherapy [13]. Worse therapy outcomes in OC patients may also be related to IL-6-mediated inhibition of differentiation and maturation of dendritic cells [20]. It has been also reported that IL-6 has the ability to upregulate the expression of programmed death ligand 1 (PD-L1) in tolerogenic antigen-presenting cells by elevating signal transducer and activator of transcription 3 (STAT3) [21]. IL-6 is known to stimulate tumor infiltration by macrophages in ovarian tissue, and this phenomenon is associated with worse prognosis of OC patients [17].

The understanding of inflammatory mechanisms in ovarian cancer is pivotal for the selection of a biomarker that allows the differentiation between malignant and benign tumors, monitoring the progression of the disease, and identification of patients that will respond to implemented treatment. Understanding how the composition of the TME changes during cancer development and progression is a prerequisite for projecting therapeutic strategies able to tackle the tumor at a specific evolutionary stage which is important for the selection of the most suitable therapy [22].

## 2. Aim of the Study

To the best of our knowledge, the IL-6 assessment in patients with various clinical manifestations of ovarian cancer has not been comprehensively described in the literature. The data regarding the prognostic value of IL-6 in ovarian cancer are inconclusive as well. Therefore, our study is aimed at evaluating the profile of IL-6 in both the plasma and peritoneal fluid of patients with ovarian cancer and benign ovarian tumors and in the plasma of healthy donors. In the next step, we examined the relationship between the levels of IL-6 and the percentage of PD-L1/PD-L2 positive CD45^+^CD14^+^ inflammatory cells in three different OC microenvironments: peripheral blood (PB), peritoneal fluid (PF), and tumor tissue (TT) and their clinical and prognostic relevance in ovarian cancer patients.

## 3. Materials and Methods

### 3.1. Patients, Ethic Statement, and Standard Protocol Approvals

A total number of 119 pretreatment women were enrolled to the study, including 31 patients with benign ovarian tumors (with serous cystadenoma), 10 healthy women, and 78 patients with epithelial ovarian cancer whose diagnosis had been confirmed by pathological reports. All the tumors were staged according to the International Federation of Gynecologists and Obstetricians (FIGO classification) [4]. The tumors were graded and classified according to the Silverberg grading system by two independent gynecological pathologists [5]. The tumor type was determined according to the Kurman and Shih classification [6]. Patients that had undergone radical treatment at the I Chair and Department of Oncological Gynaecology and Gynaecology (Independent Public Clinical Hospital No. 1, Medical University of Lublin, Poland) were enrolled to the study. The exclusion criteria for the study cohort included a history of previous malignancies, chemotherapy, or radiation therapy prior to surgery as well as allergic, autoimmune, and infectious diseases. Data, e.g., the overall survival (OS) of the patients, were obtained from the Document Personalization Center of the Ministry of Internal Affairs and Administration, Department of Protection of Confidential Information in Warsaw.

Written informed consent was obtained from all patients. The research received approval of the Bioethics Committee at the Medical University of Lublin (KE-0254/280/2015).

The control group included 10 healthy donors at the age of 21-51 (median 29 years). The PB of the control group was obtained from the Regional Centre of Blood Donation and Blood Treatment in Lublin. Every patient signed written consent for participation in the study, and the experiments were conducted in accordance with the Declaration of Helsinki. The clinical characteristics of the OC patients are summarized in Table 1.

### 3.2. Isolation of Mononuclear Cells (MNCs)

Ten milliliters (ml) of peripheral blood was collected before the surgical procedure into heparinized tubes (Sarstedt, Germany) and immediately processed. Peritoneal fluid (10 ml) and small pieces of tumor tissue (~1cm^3^) from nonmargin areas and without necrotic changes were collected aseptically during the operation.

PB and PF specimens were centrifuged (1500 rpm/10 min) to obtain plasma and rendered cell-free fluids. The plasma and supernatant of PF were immediately stored at -80°C until analysis with enzyme-linked immunosorbent assay (ELISA) tests.

For isolation of tumor-infiltrating mononuclear cells, freshly resected tumor tissue was minced with scissors into 2-4 mm pieces and processed using a modified protocol from a tissue dissociation kit with a gentle MACS dissociator (MiltenyiBiotec, Germany). The tumor tissue was placed into a gentle MACS C tube containing 5 ml of dissociation medium. Next, the samples were processed using the Tumor Dissociation Kit, (Catalog No. 130-095-929, MiltenyiBiotec, Germany) following manufacturer's instructions to obtain a single-cell suspension. The resulting material was filtered through a 70 mm mesh filter. Mononuclear cells from PB, PF, and tumor tissue were isolated by density gradient centrifugation at 700 x g with Gradisol L (*d* = 1,077 g/cm^3^, Aqua Medica, Poland) at room temperature for 20 minutes as previously described in details [23]. Before layering onto the density gradient, PB was diluted 1 : 1 with phosphate-buffered saline (PBS, PAA Laboratories GmbH, Austria). Subsequently, interphase cells were collected and washed twice in PBS (700 x g/5 min). The viability of the MNCs obtained was determined by trypan blue staining, and it was always >95%. Viable cells were quantified in a Neubauer chamber.

### 3.3. Flow Cytometry Analysis

**Figure 1:**
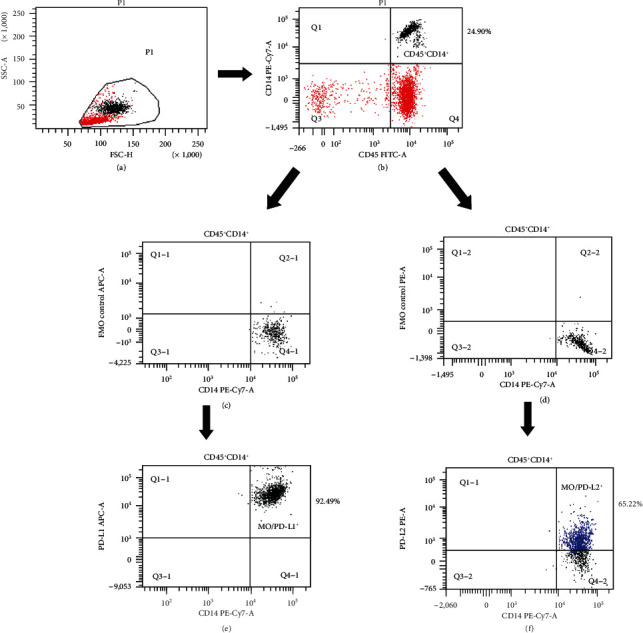
Flow cytometry analysis of CD45^+^CD14^+^ cells expressing PD-L1 or PD-L2. An acquisition gate was established based on FSC and SSC that included mononuclear cells. A population P1 was drawn around MNCs (a). Next, the P1 gated events were analyzed for CD45 FITC and CD14 Pe-Cy7 staining and positive cells (CD45^+^CD14^+^) were gated (region Q2) (b). The final dot plots CD45^+^CD14^+^ expressing (d) PD-L1 or (f) PD-L2 versus FMO control were established by combined gating of events using population P1 and region Q2-1 or Q2-2. The number in the upper right quadrant on the dot plots (d, e) represents the percentage of CD45^+^CD14^+^ expressing (d) PD-L1 or (e) PD-L2.

MNCs isolated from PB, PF, and tumor were incubated with 10 *μ*l of FcR Blocking Reagent (Miltenyi Biotec, Germany) for 10 minutes in the dark to eliminate nonspecific staining and stained for the flow cytometry analysis. Briefly, 100 *μ*l of cells (1 × 10^6^ cells per tube) were incubated in the dark with conjugated anti-human monoclonal antibodies (mAbs) for 20 min at room temperature. Next, the samples were washed twice by adding 2 ml FACS buffer (2% FBS in PBS) to each tube. The following combination of mAbs was used: anti-CD45 FITC (BD Pharmingen, Catalog No. 555482; 20 *μ*l/sample), CD14 PE-Cy7 (BD Pharmingen, Catalog No. 557742; 5 *μ*l/sample), anti-PD-L1 APC (Biolegend, Catalog No. 329708; 5 *μ*l/sample), and anti-PD-L2 PE (Biolegend, Catalog No. 329606; 5 *μ*l/sample). After staining, the cells were resuspended in 2 ml FACS buffer (autoMACS Running Buffer, Miltenyi Biotec, Germany) and the surface expression of PD-L1 or PD-L2 molecules in MO/MA was evaluated using a BD FACSCanto I flow cytometer (BD Biosciences, USA). The flow data were analyzed in the FACSDiva software (BD Biosciences, USA). The compensation control was performed with a BD CompBeads set (BD Biosciences) according to the manufacturer's instruction. Fluorescence minus one (FMO) controls were used as negative controls. The percentage of CD45^+^CD14^+^PD-L1^+^ and CD45^+^CD14^+^PD-L2^+^ cells was analyzed by flow cytometry (FACSCanto I, Becton Dickinson, USA). For each analysis, 100,000 events were acquired and analyzed using the FACSDiva software. Dot plots, illustrating the flow cytometry analysis of CD45^+^CD14^+^ cells expressing PD-L1 or PD-L2, are shown in Figure 1.

### 3.4. ELISA

IL-6 levels in the plasma and PF were determined using an enzyme-linked immunosorbent assay (Human IL-6 Quantikine Colorimetric Sandwich ELISA kit, Catalog No. D6050, R&D Systems, Minneapolis, Minnesota, USA) according to the manufacturer's protocol. Plate absorbance was read on an ELX-800 plate reader at wavelength 450 nm and analyzed using the Gen5 software (BioTek Instruments, USA) for acquisition and data analysis. Concentrations of IL-6 (pg/ml) were calculated by interpolation from a standard curve. All samples were assayed in duplicate. The sensitivity of the IL-6 ELISA kit was less than 0.70 pg/ml.

### 3.5. Statistical Analysis

The statistical analysis of the results was conducted using Statistica 12.0 PL. The Wilcoxon paired test was used to compare the results from PB, PF, and tumor tissue. The Mann-Whitney *U* test was applied to the results of the statistical comparison between the groups (control/benign/OC). Relationships between two parameters were investigated using Spearman's rank correlation test. The data are presented as medians, minimum, and maximum. The probabilities of overall survival (OS) were estimated using the Kaplan–Meier method, and differences in the survival curves were calculated using the logrank test; a *p* value less than 0.05 was considered statistically significant.

## 4. Results

### 4.1. Concentration of IL-6 in the Plasma of Patients with Ovarian Cancer, in the Group with Benign Ovarian Tumors, and in the Control Group

**Figure 2:**
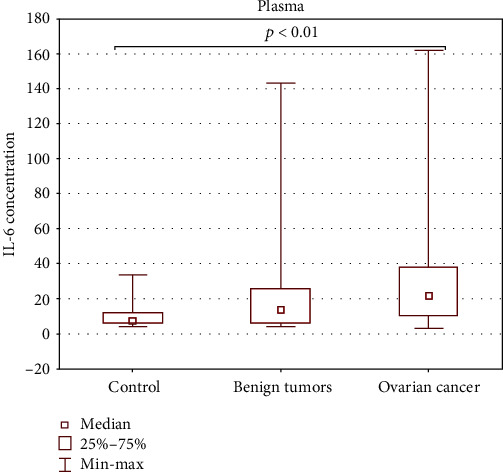
Concentration of IL-6 in the plasma of patients with ovarian tumors and healthy donors.

The highest concentration of IL-6 was detected in the plasma of ovarian cancer patients, and it was significantly higher (*p* < 0.01) than in the control group. The results are presented in Table 2 and in Figure 2.

### 4.2. Concentration of IL-6 in the Peritoneal Fluid of Patients with Ovarian Cancer and in the Group with Benign Ovarian Tumors

**Figure 3:**
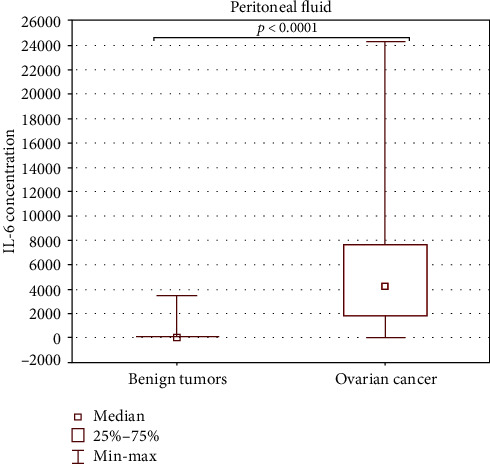
Levels of IL-6 (pg/ml) in the peritoneal fluid of patients with ovarian cancer and in the group with benign ovarian tumors.

The concentration of IL-6 in the peritoneal fluid of OC patients (median 4198.18, min-max. 29.01-24310.80 pg/ml) was significantly higher (*p* < 0.0001) than in the group with benign ovarian tumors (median 56.55, min-max. 11.70-3479.64 pg/ml). The results are presented in Figure 3.

### 4.3. Concentration of IL-6 in the Plasma and Peritoneal Fluid of Patients with Different FIGO Stages, Grades, and Kurman-Shih Types of Ovarian Cancer

The characteristics of cancer patients and the associated plasma and peritoneal fluid IL-6 levels are presented in Table 3.

The plasma IL-6 levels did not differ significantly (*p* > 0.05) between the different FIGO stages, grades, or Kurman-Shih types of ovarian cancer.

Interestingly, we detected significantly higher accumulation of IL-6 in the PF samples from patients with grade 3 of ovarian cancer than those with grade 2 (*p* < 0.001).

The PF IL-6 levels differed between the different FIGO stages or Kurman-Shih types of ovarian cancer; however, the difference was not statistically significant (*p* > 0.05).

### 4.4. Assessment of the Relationship between the IL-6 Level in Plasma and PF and the Clinical Data of OC Patients

There was no significant correlation between the plasma IL-6 concentration and the FIGO stage, grade, Kurman-Shih type of ovarian cancer patients, and the level of Ca125 (*p* > 0.05).

There was a statistically significant relationship between the IL-6 concentration in PF and the FIGO stage and the Ca125 level (R Spearman 0.352, *t*(*N* − 2) 3.281, and *p* = 0.001 and R Spearman 0.294, *t*(*N* − 2) 2.265, and *p* = 0.02, respectively). There was no significant correlation between the IL-6 level in PF and the grade or Kurman-Shih type of ovarian cancer patients (*p* > 0.05). There was a relationship between the PF levels of IL-6 and BMI of the OC patients (R Spearman: 0.28; *t*(*N* − 2) 2.43; *p* = 0.01).

The plasma and ascites IL-6 concentration were higher in the group of the postmenopausal patients with OC in comparison to the premenopausal patients; however, the difference was not statistically significant (*p* > 0.05).

### 4.5. Concentrations of IL-6 in the Plasma and Peritoneal Fluid of Patients with Ovarian Cancer and Benign Ovarian Tumors

The concentrations of IL-6 both in the patients with ovarian cancer and in the patients with benign ovarian tumors were significantly higher (*p* < 0.0001) in the peritoneal fluid than in the plasma (median 4198.18 vs. 21.72 pg/ml and 56.55 vs. 13.65 pg/ml, respectively).

### 4.6. Distribution of CD45^+^CD14^+^ MO/MA in Malignant and Nonmalignant Ovarian Tumor Microenvironments

**Figure 4:**
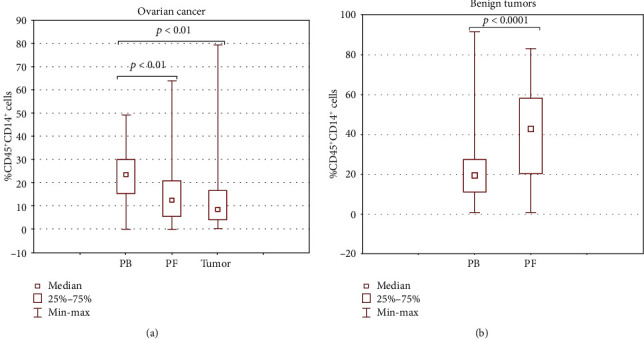
Distribution of CD45^+^CD14^+^ MO/MA in the (a) malignant and (b) nonmalignant ovarian tumor microenvironment.

Next, we asked a question whether there were differences between the distribution of MO/MA cell populations in the three different environments (PB, PF, and TT) of the OC patients and (PB and PF) of the patients with benign ovarian tumors. We observed a significant disparity in the distribution of MO/MA in both malignant and nonmalignant ovarian tumors in the examined TMEs (Figures 4(a) and 4(b)).

We observed higher accumulation of MO/MA in PB (median 23.79%) than in PF (median 12.66%) and tumor (median 8.95%) of patients with OC (Figure 4(a)). In contrast, the percentage of MO/MA in the patients with benign ovarian tumors was significantly higher in PF (median 42.93%) than in PB (median 19.52%) (Figure 4(b)).

Interestingly, the percentage of MO/MA in the peritoneal fluid of the OC patients was significantly lower (*p* < 0.0001) than in the group with benign ovarian tumors (median 12.66% vs. 42.93%, Figures 4(a) and 4(b)). There was no such difference in the peripheral blood (*p* > 0.05).

### 4.7. Expression of PD-L1/PD-L2 on Inflammatory/Immune CD45^+^CD14^+^ Cells in PB, PF, and Tumor Tissue of OC Patients

**Figure 5:**
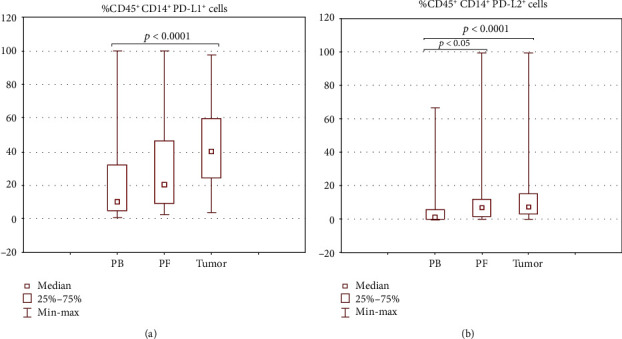
Percentage of CD45^+^CD14^+^ cells with expression of (a) PD-L1 and (b) PD-L2 in the TMEs of OC patients.

We also found a significant disparity in the distribution of PD-L1/PD-L2 positive monocytes/macrophages in the three examined TMEs, i.e., peripheral blood, PF, and tumor tissue in the OC patients (*n* = 50). The highest percentage of PD-L1^+^ and PD-L2^+^ macrophages was detected in the tumor tissue, and it was significantly higher (*p* < 0.0001) than in PB (median 40.43% vs. 10.41% for PD-L1^+^ cells and 7.38% vs. 1.43% for PD-L2^+^ cells) (Figures 5(a) and 5(b)). The percentage of CD45^+^CD14^+^PD-L2^+^ cells was significantly higher (*p* < 0.05) in the peritoneal fluid than PB of the OC patients (median 7.11% vs. 1.43%).

We showed no significant disparity in the distribution of PD-L1 and PD-L2 positive monocytes/macrophages in the blood, PF, and tumor tissue in the different clinicopathological features of the OC patients.

### 4.8. Peritoneal Fluid IL-6 Levels Are Increased in Ovarian Cancer Patients and Correlate with the Percentage of CD45^+^CD14^+^ Inflammatory Cells

**Figure 6:**
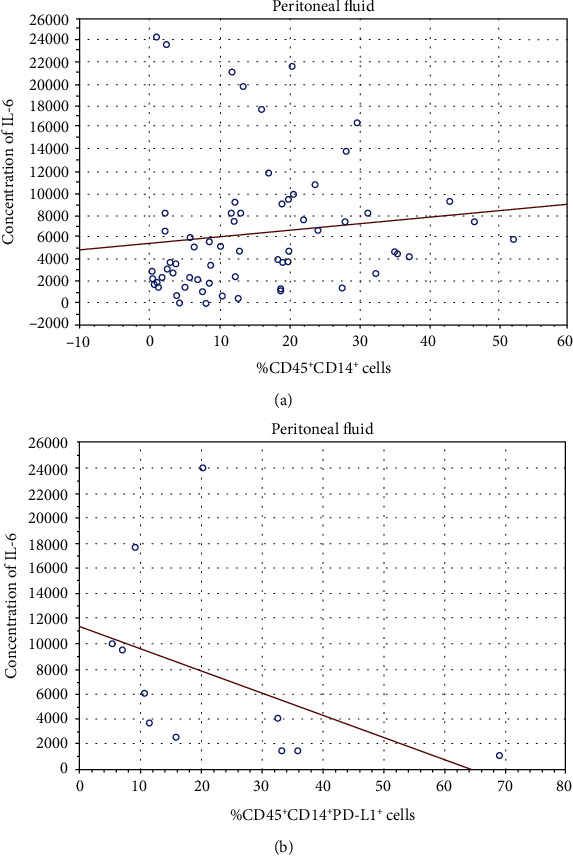
Relationship between the IL-6 levels and the percentage of (a) CD45^+^CD14^+^ and (b) CD45^+^CD14^+^ PD-L1^+^ cells in PF of OC patients.

Next, we asked whether there was a relationship between the levels of IL-6 and the percentage of MO/MA cells in the studied group of patients. We distinguished significantly positive correlations between the level of IL-6 and the percentage of CD45^+^CD14^+^ cells in PF of the OC patients (R Spearman: 0.33; *t*(*N* − 2) 2.81; *p* = 0.006, Figure 6). We observed no correlations between the level of IL-6 in the plasma and the percentage of MO/MA cells in PB of the OC patients (*p* > 0.05). There were no correlations between the level of IL-6 in both the plasma and PF and the percentage of MO/MA cells in the group of benign ovarian tumors (*p* > 0.05).

There was also a relationship between the percentage of CD45^+^CD14^+^ PD-L1^+^ cells and the level of IL-6 in PF (*n* = 11) of the OC patients (R Spearman: -0.73; *t*(*N* − 2) -3.26; *p* = 0.009). We observed no correlations between the level of IL-6 in the plasma and the percentage of CD45^+^CD14^+^ PD-L1^+^/PD-L2^+^ cells in PB of the OC patients (*p* > 0.05).

### 4.9. Clinical Parameters and Association with OC Patient Survival

**Figure 7:**
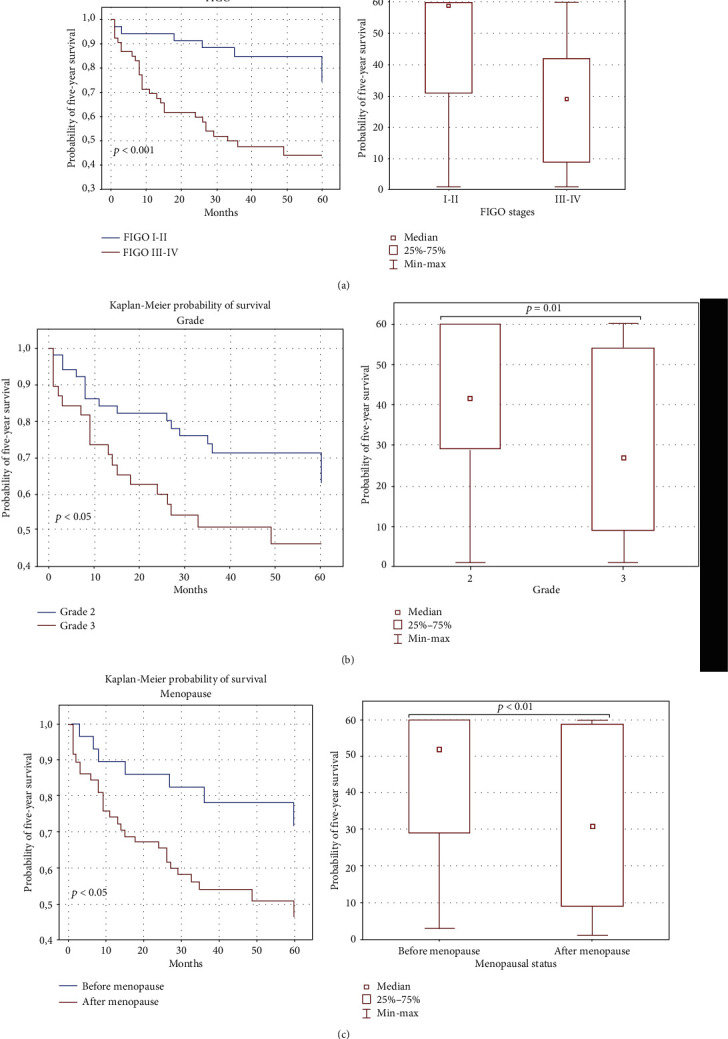
Relationship between clinical parameters and 5-year survival of OC patients.

In the next step, we evaluated the relationship between clinical parameters and 5-year survival of the OC patients. We found that patients diagnosed with advanced disease (FIGO stages III and IV) had shorter OS than those with FIGO stages I and II diseases (median 33 vs. 60 months), and the difference reached statistical significance (*p* < 0.001). Additionally, patients diagnosed with tumor grade 3 had shorter OS than those with grade 2 of OC (median 34 vs. 51.5 months). Type of OC according to the Kurman and Shih classification had no statistically significant association with patients' survival (*p* > 0.05). The premenopausal patients had median OS of 60 months versus 39 months in the postmenopausal ones (*p* < 0.01). Significant parameters for these correlations are shown in the Kaplan–Meier plots (Figure 7).

**Figure 8:**
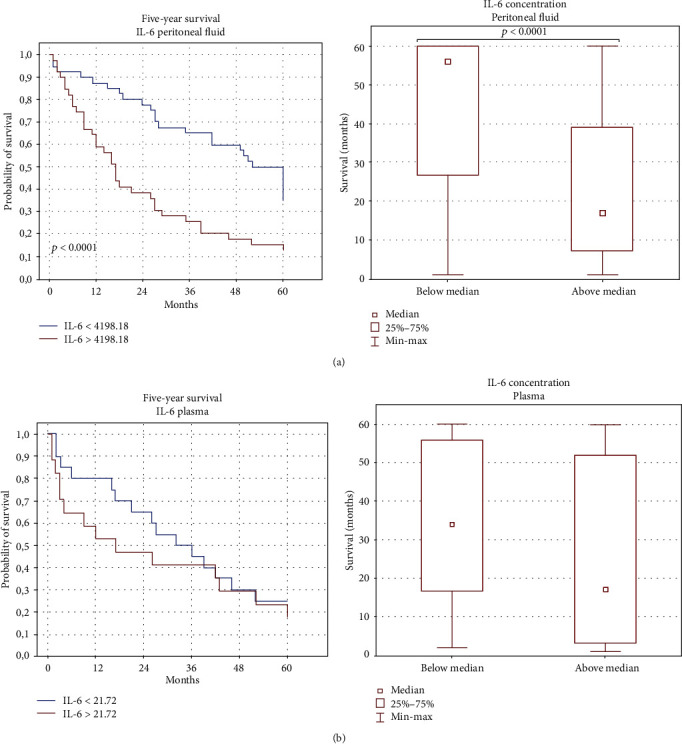
Relationship between the (a) ascites and (b) plasma IL-6 levels and 5-year survival of OC patients.

Similar examination was performed to determine the OS estimates as a function of the IL-6 levels in the plasma and PF of the OC patients. Interestingly, those with the higher IL-6 level in PF had shorter 5-year survival than the patients with the lower IL-6 concentration (median 17 vs. 56 months, *p* < 0.05) (Figures 8(a) and 8(b)).

Also, patients with the higher IL-6 concentration in the plasma had shorter OS (median 16 vs. 33 months) than those with the lower IL-6 level; however, the difference was not significant (*p* > 0.05).

**Figure 9:**
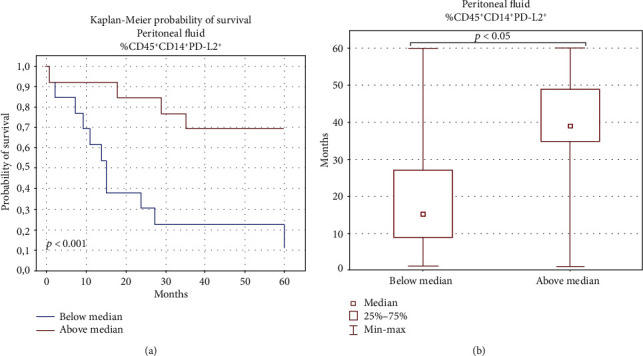
Relationship between CD45^+^CD14^+^ cells with PD-L2 expression in the PF and 5-year survival of OC patients.

Surprisingly, the OC patients with the higher percentage of PD-L2^+^ macrophages in PF had longer OS (median 38 vs. 15 months) than those with the lower percentage of these cell populations (Figure 9).

## 5. Discussion

From the immunological point of view, TME is essential in OC pathogenesis. The TME features preclude induction of effective anticancer response by immune system cells. Numerous studies are focused on elucidation of the mechanism of the dysfunction by targeting regulatory T cells (Tregs), myeloid-derived suppressor cells (MDSCs), macrophages, immune checkpoints (ICPs), chemokines, and cytokines [1, 2, 22]. Determination of the disease background and reliable prediction factors is essential for the development and implementation of targeted therapy for treatment and improvement of patient quality of life.

In our research, we investigated the concentration of IL-6 in different OC environments, e.g., PB plasma and peritoneal fluid (PF). In addition, we determined the profile of IL-6 in both plasma and PF of patients with benign ovarian tumors and healthy donors. In the next step, we examined the clinical and prognostic relevance of IL-6 and the percentage of PD-L1/PD-L2 positive monocytes/macrophages (MO/MA) in terms of their clinical and prognostic relevance in ovarian cancer patients.

In the present study, we detected accumulation of IL-6 in the plasma of OC patients in comparison to the control group. Additionally, the IL-6 level in the peritoneal fluid of the OC patients was significantly higher than in the plasma. Moreover, the level of IL-6 in the PF of the OC patients was higher compared to the patients with benign ovarian tumors. Similarly, Block et al. [24] have demonstrated higher IL-6 plasma levels in OC patients compared to a group with benign ovarian tumors. The results are consistent with findings reported by another research team, who detected a higher level of IL-6 in the serum of OC patients [8]. Similarly, Chudecka et al. [25] and Lane et al. [26] show higher IL-6 levels in the serum of OC patients compared to patients with benign ovarian tumors. The authors have suggested that determinations of IL-6 levels in PF can be useful in the differentiation between malignant and benign ovarian tumors [25, 26]. Our findings are consistent with the results reported by Giuntoli et al. [27], who demonstrated substantially higher levels of IL-6 in ascites than in serum. As in our study, another research team detected higher IL-6 levels in ascites from high-grade serous (HGCS) OC patients compared with serum [8]. In their study, Lima et al. [28] found higher IL-6 values in OC patients compared to those with nonmalignant tumors. Similarly, Dalal et al. [29] showed higher levels of IL-6 in ascites of OC patients compared with controls.

The accumulation of IL-6 in the peritoneal fluid of the OC patients demonstrated in our study may be of importance for the prognosis of an adverse course of the disease. According to the literature data, IL-6 may stimulate the growth of neoplastic cells in an autocrine manner. It has been shown that TME factors are capable of activating macrophages, which leads to the synthesis of TNF-*α* and IL-6. This promotes the ability of TME and tumor to generate metastases [11]. Moreover, IL-6 has been found to intensify cell proliferation, invasion, and angiogenesis. An elevated level of IL-6/IL-6R has been demonstrated to increase the expression of VEGF, which is the best-known stimulator of angiogenesis [13]. As suggested by Soochi et al. [30], the presence of IL-6 in the peritoneal fluid intensifies the invasion and migration of ovarian cancer cells. The elevated levels of IL-6 in the OC patients observed in our studies may therefore contribute to tumor progression and impede immunotherapy [10, 31–33]. The findings reported in the present study and by other authors imply local IL-6 synthesis at the site of the ongoing inflammatory process. The likely sources of IL-6 are neutrophils, monocytes/macrophages, fibroblasts, lymphocytes, neoplastic cells, and mesothelial cells [13]. Noteworthy, the level of IL-6 produced by mesothelial cells has been shown to be even 600-folds higher compared to ovarian cancer cells [34]. The increased IL-6 synthesis observed in PF may evidence the autonomy of the peritoneal environment in OC patients in whom the neoplastic process spreads. The accumulation of IL-6 in PF observed in our study may promote intensification of angiogenesis and the peritoneal dissemination. We speculate that IL-6 accumulation in PF of OC patients might be one of the important causes of the poor efficacy of implemented immunotherapy.

The IL-6 assessment in patients with various clinical manifestations of OC has not been comprehensively described in the literature. In the present study, we did not observe statistically significant differences in the plasma IL-6 levels, depending on the FIGO stage, grade, and type of OC. However, the significantly elevated levels of IL-6 in PF observed in our study in patients with poorly differentiated (G3) but not moderately differentiated (G2) OC are of interest. Moreover, elevated IL-6 levels have been found in PF of patients with advanced stages of the disease (FIGO III-IV) and those with type II of OC.

Our data indicate that high synthesis/accumulation of IL-6 in the peritoneal fluid of OC patients can be TME dependent. Similarly, Dalal et al. [29] have demonstrated significantly elevated levels of IL-6 in PF of patients with advanced vs. early stage of disease. Moreover, the authors have revealed higher IL-6 levels in the ascites of patients with high-grade OC [29]. Chudecka et al. [25] have noted higher levels of IL-6 in the peritoneal fluid of patients with advanced FIGO stages of OC. Moreover, Chechlińska et al. [35] have reported higher PF levels of IL-6 and VEGF in OC patients than in benign tumor patients, irrespective of the stage. As suggested by the authors, the diagnostic value of such determinations is relatively high for differential diagnosis of malignant and benign tumors [35]. Our observations differ from the findings presented by Lane et al. [31], who did not demonstrate a correlation between the level of IL-6 in the peritoneal fluid versus the FIGO clinical stage, histopathological differentiation, and Kurman-Shih type of OC.

Our findings revealed that the survival of the OC patients significantly depended on the IL-6 concentration in the peritoneal fluid. Interestingly, overall survival (OS) of patients with higher IL-6 levels in PF was significantly shorter (median: 17 months vs. 56 months) compared to patients with lower IL-6 levels. However, the data regarding the relationship between the IL-6 concentration and OS of OC patients are inconclusive. As demonstrated by the majority of reports, elevated levels of IL-6 correlate with shorter progression-free survival (PFS) [26, 29, 31, 36] and shorter OS [31, 37, 38]. Some other authors have not observed such a relationship [39].

It is worth stressing that the exact mechanism resulting in poor prognosis of patients with elevated IL-6 levels has not been fully elucidated. It has been suggested that high levels of IL-6 may induce resistance to chemotherapy, leading to earlier recurrences [29]. *In vitro* study findings reported by some authors indicate that IL-6 induces resistance to cisplatin via upregulation of the synthesis of antiapoptotic proteins (Bcl-2, IAPs) and downregulation of the synthesis of proapoptotic proteins (BID, BAX) [37]. As shown in another study, survival is significantly affected not only by the level of IL-6 but also the IL-6 genotype. The IL-6-174 GG genotype (versus CC or CG) has been shown to correlate with prolonged OS [38]. It has also been reported that IL-6 can stimulate tumor infiltration by macrophages, and this phenomenon is associated with worse prognosis of OC patients [17]. Moreover, it affects immune cells e.g., enhancing immunosuppression by inducing the B7-H4 expression on macrophages. Furthermore, by inhibiting the synthesis of IL-2, it decreases the activity of T lymphocytes and induces their apoptosis, enabling neoplastic cells to escape from immune control [13]. IL-6 has also been found to shift the Th1–Th2 balance towards Th2 immunity, yet the available data are divergent [31]. A study carried out in a mouse model has suggested that ovarian cancer-associated fibroblasts produce IL-6 in the OC microenvironment and induce differentiation of tumor stem cells, which contributes to postchemotherapy recurrences [39]. A recent study reported that IL-6 was involved in Jumonji C-domain family 2- (JMJD2A/KDM4-) mediated progression of ovarian cancer [9].

One of the causes of antitumor response inhibition is the expression of immune checkpoints (ICPs). In normal conditions, ICPs are essential for the maintenance of tolerance towards self-antigens [40, 41]. However, in pathological conditions, including OC, ICPs such as PD-1 and its ligand PD-L1/PD-L2 become negative regulators that may inhibit T cell functions and mediate in cancer immune escape from immune system surveillance [42, 43]. Previous clinical trials based on PD-1/PD-L1 inhibitors confirmed a favorable safety profile and persistent antitumor response only in a small group of OC patients [44, 45]. Thus, it is necessary to find predictive markers and understand the TME to implement efficient treatments for precise groups of OC patients [41, 46–48].

In present study, we analyzed the expression of ligands for programmed cell death receptor-1, i.e., PD-L1 and PD-L2, on MO/MA in OC patients in three different environments: PB, PF, and tumor (TT). The results of these analyses were correlated with the clinicopathological features of the OC patients and the level of IL-6.

Interestingly, we found differences in the PD-L1 and PD-L2 expression on CD45^+^CD14^+^ cells in the three OC environments. We detected accumulation of cells with PD-L1 expression in the tumor in comparison to PB. The percentage of CD45^+^CD14^+^PD-L1^+^ cells was higher among the OC-infiltrating cells than in PF and in PF in comparison to PB; however, these differences did not reach statistical significance.

Additionally, we detected accumulation of CD45^+^CD14^+^PD-L2^+^ cells in the tumor and PF in comparison to PB. What is interesting, the highest percentage of CD45^+^CD14^+^PD-L1^+^ and CD45^+^CD14^+^PD-L2^+^ was detected among the tumor-infiltrating cells.

Gottlieb and coworkers [46] have shown PD-L1 expression on TAMs, specifically in primary epithelial ovarian cancer and metastatic HGSC. The PD-L1 expression was higher on macrophages (74%) than on cancer cells (8%). Similarly, Qu et al. [49] and Webb et al. [50] have reported PD-L1 expression among CD68^+^ tumor-infiltrating macrophages. The presence of CD68^+^PD-L1^+^ cells in PB of OC patients has been shown as well [49]. Webb and coworkers have demonstrated that PD-L1^+^ cells often occur collectively with CD8^+^, CD4^+^, and PD-1^+^ tumor-infiltrating T cells, CD25^+^FoxP3^+^ Tregs, and other TIL populations [50]. Studies in a murine model of OC have shown high PD-L1 expression among CD45^+^ cells in PF, including TAMs [51]. Moreover, in the same model, Duraiswamy et al. [52] have reported high PD-L1 expression and moderate PD-L2 expression on cancer cells, macrophages, dendritic cells, and MDSCs. Lin and coworkers [51] have conducted studies in OC patients treated with mAbs targeted against PD-1. They have demonstrated that PD-L1 expression on APCs, including macrophages, correlates positively with clinical response in melanoma and OC patients subjected to this kind of treatment. Considering these data, the role of the PD-L1 expression on cancer cells and host immune system cells is extremely interesting for further investigations in OC patients [51].

It is well established that clinical parameters such as the FIGO stage, histological type of tumor, and menopausal status are prognostic factors for OC patients [53]. However, the relationship between immunological factors still remains unclear. Thus, we also investigated the relationship between clinicopathological parameters and immune system factors. We did not find a statistically significant correlation between the percentage of MO/MA with PD-L1 or PD-L2 expression and the clinical parameters of the patients. In turn, studies conducted by other authors and preliminary clinical trials indicate that TAMs are highly involved in the early-stage spread of OC [54, 55]. The data obtained by Silverberg [5] show that PD-L1 expression on monocytes and CD14^+^ cells in PB was significantly higher at the early stage (FIGO I) of OC in comparison to benign tumors. As reported by Silverberg [5], the percentage of PD-L1^+^CD68^+^ cells and PD-L1 expression were higher at the advanced stage of cancer (FIGO III-IV) than at the early stage (FIGO I-II) of disease.

As shown in the literature, primary epithelial OC is characterized by PD-L1 expression only on immune system cells. In recurrent disease, the expression of PD-L1 is observed on both immune system cells and cancer cells [48, 56, 57]. The analysis of platinum-sensitive recurrent OC TME indicated a higher percentage of “classically activated” macrophages M1 and CD25 ^+^Tregs in primary tumor. These results correlated with longer PFS of OC patients [46].

A study conducted by Qu et al. [49] demonstrated that both the percentage of CD68^+^PD-L1^+^ macrophages and the level of PD-L1 expression on these cells were elevated in patients with OC than in patients with benign tumors. The data on the relationship between MO/MA density and prognosis in cancer patients are divergent, considering the complexity of immunological reactions in the OC microenvironment. As indicated by some reports, tumor infiltration by TAMs is related to poor prognosis [55, 58]. Via PD-L1 expression, TAMs may lead to inactivation of T cells and decrease their capability of antitumor response [55]. On the other hand, T cells have an influence on the effector functions of TAMs. The cytokines produced by Th2 cells, i.e., IL-4 and IL-13, may change their phenotype into the immunosuppressive type (M2). Investigations of human melanoma cells show that IFN-*γ* produced by Th1 cells induces the expression of colony-stimulating factor 1 (CSF-1) and promotes tumor infiltration by M2-type TAMs [51]. However, in agreement with our results, Ojalvo et al. [48] did not find a statistically significant correlation between the density of TAMs and patient survival. In contrast, tumor infiltration by macrophages in colorectal cancer was related to favorable prognosis [59, 60].

Webb et al. [50] have reported that simultaneous presence of both PD-L1^+^ macrophages and CD8^+^ TILs is associated with improved prognosis in comparison to the presence of only CD8^+^ TILs in HGSC. Contrarily, Hamanishi et al. [61] have demonstrated that higher PD-L1 expression is related to a reduced TIL percentage and poor prognosis. However, the analysis did not reveal which cells expressed PD-L1, and the staining pattern and background staining suggested that they were rather epithelial cells than TAMs [50]. What is more, the study conducted by Chatterjee et al. [62] indicates that PD-L1 expression on inflammatory cells associated with tumor is related to shorter PFS.

The research carried out by Taube et al. has shown the presence of TILs; however, they did not observe the presence of cells with PD-L1 expression [41]. They suggest that IFN-*γ* produced by TILs is responsible for the PD-L1 expression on immune system cells and cancer cells. The lack of PD-L1 expression may indicate that the T cell response is not always equally efficient and may not lead to appearance of PD-L1 on cells [41, 50].

Accumulation of macrophages with PD-L1 expression in tumor is an indicator of the efficiency of mAb therapy targeted against PD-1 and PD-L1 in some malignancies. Patients with PD-L1 expression on 10% of macrophages responded to the implemented treatment at the response rate of 80% [63]. Interestingly, our study shows for the first time that the higher percentage of CD45^+^CD14^+^PD-L2^+^ cells in PF predicts better survival of OC patients. These and other author's results suggest that the analysis of the expression of immune checkpoints on the immune cell surface rather than on cancer cells is more relevant [62].

## 6. Conclusions

In summary, to our knowledge, this is the first research targeted at the profile of IL-6 in both plasma and PF of patients with different clinical manifestations of OC in comparison to benign tumors and healthy donors. Our results indicate that the elevated concentration of IL-6 in PF is correlated with shorter survival of OC patients. Additionally, we distinguish significantly positive correlations between the level of IL-6 and the percentage of CD45^+^CD14^+^ cells in PF and a negative relationship between the percentage of CD45^+^CD14^+^PD-L1^+^ cells and the level of IL-6 in PF of OC patients. We have shown for the first time that the higher percentage of CD45^+^CD14^+^ cells expressing PD-L2^+^ in PF is associated with beneficial survival outcomes.

Our study suggests that CD45^+^CD14^+^PD-L2^+^ cells and IL-6 may be predictive biomarkers for OC patients.

The present results are very interesting; however, due to the high molecular and clinical diversity of OC, experiments should be carried out in a group comprising more OC patients. Validation of these results is still required; nevertheless, they may be clinically relevant in simple, noninvasive, real-time monitoring of OC patients. Overall, further validation research is warranted.

## Figures and Tables

**Table 1:** Clinical characteristics of OC patients.

Clinical features	Ovarian cancer patients (*n* = 78)
Age (median), years (range)	55 (22-89)
FIGO stage, *n* (%)	
Early (I-II)	24 (31%)
I	8 (10%)
II	16 (21%)
Advanced (III-IV)	54 (69%)
III	38 (49%)
IV	16 (20%)
OC classification according to Kurman and Shih, *n* (%)	
Type I (endometrioid, serous G2, mucinous)	47 (60%)
Type II (serous G3)	31 (40%)
Grading (histological differentiation), *n* (%)	
Intermediate grade (G2)	32 (41%)
Low grade (G3)	46 (59%)
Ca125 range, median (U/ml)	1110.0 (45.71-19829.0)
BMI range, median	27.58 (19.83-49.78)

**Table 2:** Levels of IL-6 (pg/ml) in the plasma of patients with ovarian tumors and healthy donors.

Group	Concentration of IL-6 (pg/ml) in the plasma		
	Median	Minimum	Maximum
Ovarian cancer (*n* = 78)	21.72^∗^	2.98	162.15
Benign tumors (*n* = 31)	13.65	3.90	143.44
Control group (*n* = 10)	6.82	3.90	33.15

^∗^ *p* < 0.01 in relation to the control group.

**Table 3:** Levels of IL-6 (pg/ml) in the plasma and PF of patients with different FIGO stages, grades, and Kurman-Shih types of ovarian cancer.

Concentration of IL-6 (pg/ml) in ovarian cancer patients (*n* = 78)		Plasma			Peritoneal fluid		
		Median	Minimum	Maximum	Median	Minimum	Maximum
FIGO stage	I-II (*n* = 24)	14.82	2.98	89.17	2745.37	29.01	11947.10
	III-IV (*n* = 54)	22.18	3.45	162.15	5254.85	115.27	24310.80
Grade	G2 (*n* = 32)	27.69	3.45	107.92	3135.68	464.75	19882.90
	G3 (*n* = 46)	17.87	2.98	162.15	^∗^4816.36	29.01	24310.80
Kurman and Shih type	I (*n* = 47)	21.72	2.98	162.15	3853.14	29.01	24037.60
	II (*n* = 31)	18.60	6.69	121.69	4701.35	115.27	24310.80

^∗^ *p* < 0.001 in relation to patients with grade 2 (G2) of ovarian cancer.

## Data Availability

The data used to support the findings of this study are included within the article.
